# Back to Where It Was First Described: Vectors of Sylvatic Yellow Fever Transmission in the 2017 Outbreak in Espírito Santo, Brazil

**DOI:** 10.3390/v14122805

**Published:** 2022-12-15

**Authors:** Luciana Matos de Abreu Stanzani, Monique de Albuquerque Motta, Rafael Santos Erbisti, Filipe Vieira Santos de Abreu, Agostinho Cardoso Nascimento-Pereira, Anielly Ferreira-de-Brito, Maycon Sebastião Alberto Santos Neves, Gláucio Rocha Pereira, Glauber Rocha Pereira, Claudiney Biral dos Santos, Israel de Souza Pinto, Creuza Rachel Vicente, Álvaro Adolfo Faccini-Martínez, Karina Ribeiro Leite Jardim Cavalcante, Aloísio Falqueto, Ricardo Lourenço-de-Oliveira

**Affiliations:** 1 Centro de Ciências da Saúde, Universidade Federal do Espírito Santo, Vitória 29040-090, ES, Brazil; 2 Laboratório de Mosquitos Transmissores de Hematozoários, Instituto Oswaldo Cruz, Fundação Oswaldo Cruz, Rio de Janeiro 21040-900, RJ, Brazil; 3 Departamento de Estatística, Universidade Federal Fluminense, Niterói 24210-201, RJ, Brazil; 4 Laboratório de Comportamento de Insetos, Instituto Federal do Norte de Minas Gerais, Campus Salinas, Salinas 39560-000, MG, Brazil; 5 Instituto Federal de Educação, Ciência e Tecnologia do Pará, Campus Itaituba, Itaituba 68183-300, PA, Brazil; 6 Research Institute, Fundación Universitaria de Ciencias de la Salud—FUCS, Bogotá 110911, Colombia; 7 Servicios y Asesorías en Infectología—SAI, Bogotá 110011, Colombia; 8 Coordenação Geral de Laboratórios de Saúde Pública, Secretaria de Vigilância em Saúde, Ministério da Saúde, Brasília 70000-000, DF, Brazil

**Keywords:** yellow fever, Culicidae, arbovirus, vector-borne-diseases, Atlantic Forest, *Haemagogus*, *Sabethes*, *Aedes*, *Shannoniana*

## Abstract

Evidence of sylvatic yellow fever was first reported in Atlantic Forest areas in Espírito Santo, Brazil, during a yellow fever virus (YFV) outbreak in 1931. An entomological survey was conducted in six forest sites during and after an outbreak reported ~80 years after the last case in the area. Among 10,658 mosquitoes of 78 species, *Haemagogus leucocelaenus,* and *Hg. janthinomys*/*capricornii* were considered the main vectors as they had a relatively high abundance, co-occurred in essentially all areas, and showed high YFV infection rates. *Sabethes chloropterus*, *Sa. soperi*, *Sa. identicus*, *Aedes aureolineatus*, and *Shannoniana fluviatilis* may have a secondary role in transmission. This is the first report of *Sa. identicus*, *Ae. aureolineatus*, and *Sh. fluviatilis* infected with YFV. Our study emphasizes the importance of entomological monitoring and maintenance of high vaccination coverage in receptive areas to YFV transmission.

## 1. Introduction

Yellow fever is an acute infectious disease of humans and non-human primates (NHPs). It is endemic to tropical and sub-tropical Africa and Latin America and has recently emerged in non-endemic areas of these continents. Its etiologic agent is yellow fever virus (YFV), a positive-sense single-stranded RNA arbovirus of the genus *Flavivirus* (*Flaviviridae*), which is transmitted between vertebrates by competent female mosquito vectors [1,2].

The transmission of YFV can occur through two main epidemiological cycles: urban and sylvatic. In the urban cycle, humans are the only vertebrate hosts, and the domestic mosquito *Aedes* (*Stegomyia*) *aegypti* is the vector. In Brazil, this cycle has not occurred since 1942 [3,4]. In the sylvatic cycle, arboreal mosquitoes of the genera *Haemagogus* and *Sabethes* are the most important vectors of YFV in the Americas. The amplifier vertebrate hosts are NHPs, and humans are infected when they approach or enter an enzootic forest [5,6,7].

Soper et al. (1933) reported the first human cases of sylvatic yellow fever in areas “without *Aedes aegypti*” in the Canaan Valley—an Atlantic Forest zone in Santa Teresa (ST) [8], state of Espírito Santo (ES)—during a large YFV epidemic that affected southeastern Brazil from 1931 to 1940 [3]. Intermittent epizootic waves of YFV have subsequently occurred, but no YFV infections have been detected in ES since the early 1930s [2].

In 2015, Brazil recorded increased YFV activity, with the detection of successive epizootic and human cases in the midwestern and southeastern regions. This YFV epizootic wave spread into Atlantic Forest areas where the disease had not been recorded for almost eight decades, and vaccination was not recommended [2]. This culminated in the most severe outbreak of yellow fever in the recent history of Brazil.

**Figure 1:**
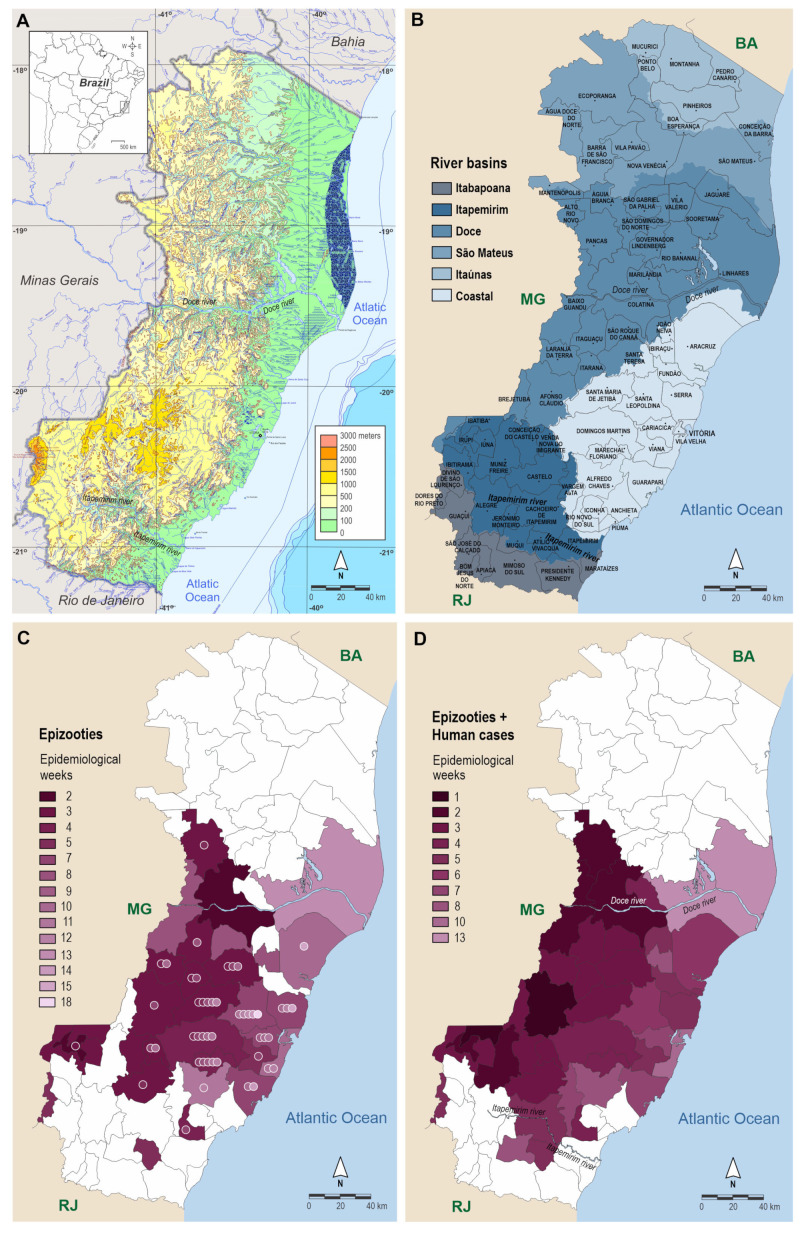
Spatiotemporal spread of YFV in the state of Espírito Santo (ES) during the 2017 outbreak. (**A**) Relief of ES. (**B**) Main river basins through which the virus entered the state in January 2017, including the Doce River Basin (to the North) and Itapemirim River Basin (to the South). (**C**,**D**) spatiotemporal spread of YFV by epidemiological week (EW). Each municipality is highlighted with the colour corresponding to the EW in which the YFV infection was first registered. The first recorded cases are in a darker tone, and the tones lighten over time. In C, the spatiotemporal evolution of epizootics registered in the Notifiable Diseases Information System (“Sistema de Informação de Agravos de Notificação: SINAN”). The circles within each municipality represent the number of EWs for which there were YFV epizootic records. In (**D**), the first records of YFV in each municipality for both epizootic (SINAN) and human cases (“Sistema de Gerenciador de Ambiente Laboratorial: GAL”). Neighboring states: Bahia (BA), Minas Gerais (MG), and Rio de Janeiro (RJ).

In this outbreak, two YFV lineages spread by different routes in southeastern Brazil: the YFV_MG/SP_ spread from the southwestern area of Minas Gerais (MG) to São Paulo (SP) state and the YFV_MG/ES/RJ_ that moved to the eastern portion of MG in the transition area between the Cerrado (a savannah-like bioregion) and Atlantic Forest in December 2016 and reached the west of ES in January 2017 via the Itapemirim and Doce River basins and moved eastward to the central and coastal municipalities (Figure 1). Four states in southeast Brazil were affected in the same year, including MG, ES, SP, and Rio de Janeiro [7,9,10,11]. More than 1600 epizootics, affecting mainly howler monkeys (*Alouatta guariba clamitans*) and marmosets (*Callithrix* spp.), 1900 human cases, and 593 human deaths were reported up to April 2018 [12,13,14,15].

The highest incidence of yellow fever occurred in ES (4.85 human cases per 100,000 inhabitants), with most cases recorded in individuals with forest-related occupations. Of the 78 ES municipalities, 34 were affected, with 196 epizootics, 179 laboratory-confirmed human infections, and 58 human deaths recorded between January and May 2017. No circulation of YFV has been detected since then [16].

A poor understanding of sylvatic transmission dynamics in these affected areas, considered the YFV-free zone for decades in ES, hinders the evaluation of the viral distribution and timely determination of high-risk areas. Brazilian epidemiological surveillance agencies recommend entomological investigation as an effective strategy to supplement YFV surveillance and prophylaxis [12]. Specifically, in the case of ES, where YFV re-emerged after eight decades without transmission, there was an absolute lack of information on whether the vector species would be the same as that implicated in transmission in the Amazon, Cerrado, or other countries or territories touched in the recent outbreaks. Also, it was unknown if there would be a local synergistic combination of vector species, including bridge vectors, that could promote a spill-over to a peri-urban or urban transmission. Accordingly, using entomological surveys, this study investigated the sylvatic YFV transmission in ES shortly after the first confirmed cases of viral circulation in January 2017. We aimed to (1) determine the richness, diversity, abundance, and distribution of mosquito species and identify local vectors; (2) characterize the vertical distribution of mosquitoes in forests; and (3) compare the diversity, abundance, and infection rates of important vectors during and after the epidemic peak. Sampling was conducted in six distinct forest areas to represent the different microregions affected by the YFV outbreak. The ST municipality, where transmission of sylvatic yellow fever was first described [8], was also included.

## 2. Materials and Methods

**Figure 2:**
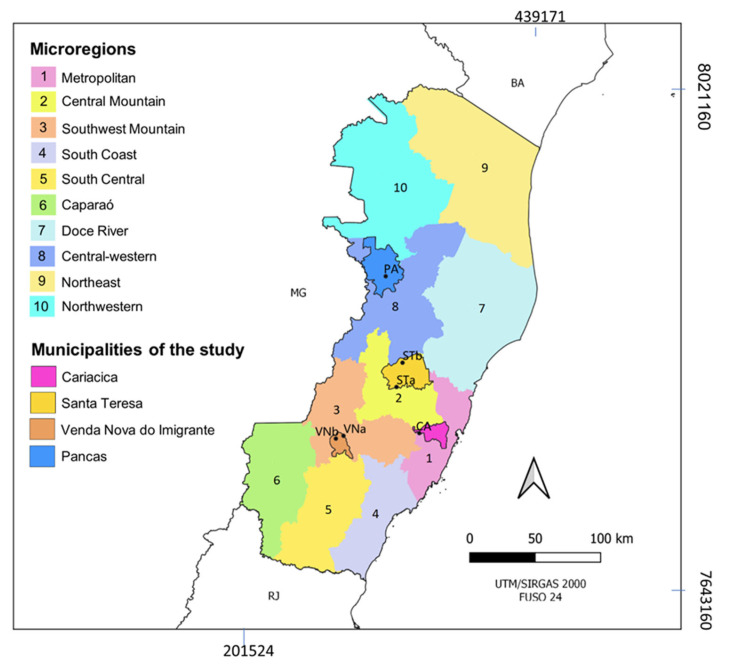
Microregions in Espírito Santo highlighting the municipalities and study sites: Venda Nova do Imigrante (VNa, −41.111229 W, −20.302705 S and VNb, −41.167397 W, −20.321149 S), Santa Teresa (STa, −40.719653 W, −19.971218 S and STb, −40.673782 W, −19.804625 S), Pancas (PA, −40.789924 W, −19.208688 S), and Cariacica (CA, −40.556154 W, −20.289890 S). Neighboring states: Bahia (BA), Minas Gerais (MG), and Rio de Janeiro (RJ).

**Figure 3:**
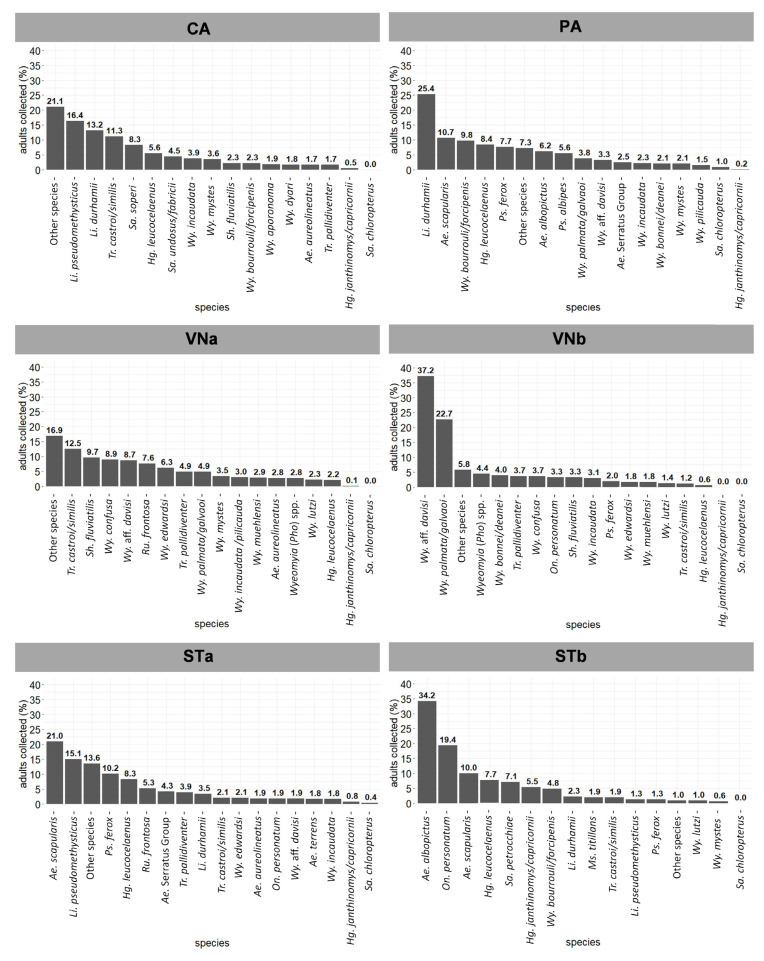
Most common mosquito species in adult sampling 1 in Cariacica (CA), Pancas (PA), Venda Nova do Imigrante (VNa and VNb), and Santa Teresa (STa and STb), Espírito Santo, February 2017.

This study was conducted at six sites located in four municipalities of ES: ST, Venda Nova do Imigrante (VN), Pancas (PA), and Cariacica (CA) (Figure 2). The criteria for selecting the study sites were as follows: (1) confirmed YFV epizootic (event in which one or more NHPs are found dying or dead) or human infections during the 2017 outbreak; localization (2) in distinct river basins (Figure 1); (3) microregions with different altitudes and environmental conditions (Figure 2); and (4) the Canaan Valley in ST, where sylvatic yellow fever was first described early in the 1930′s, for the reassessment of local vector transmission. Two sites located at different altitudes were selected in VN and ST: Lavrinhas (VNa, 930 m above sea level) and Alto Bananeiras (VNb, 1140 m) in VN, and Alto Caldeirão (STa, 870 m) and São João de Petrópolis (STb, 175 m) in ST. The sites in PA and CA were Lajinha (170 m) and São Paulo de Viana (610 m), respectively (Figure 2).

VNa and VNb are associated with large forest fragments in the southwest mountain region of the Itapemirim River Basin. They have a sloping relief, cold climate, mean annual temperature (MAT) of 18.6 °C and mean annual precipitation (MAP) of 1364 mm. STa and STb are in the central mountain region of the southern channel of the Rio Doce Basin, in areas heavily exploited for agriculture, with small forest fragments. STa is characterized by a mountainous relief, mild temperatures (MAT 19.5 °C), and a MAP of 1492 mm. STb is located at a low altitude in a wide undulating valley with a warm (MAT 24.4 °C) and dry (MAP 1045 mm) climate. PA is in the central-western region of the northern channel of the Rio Doce Basin. Its rugged relief is marked by a large rocky area of high elevation with forests restricted to the valleys. Its climate is warm (MAT 24.5 °C) and dry (MAP 1192 mm). CA is in the metropolitan region of Vitória (the state capital) in the east, connecting the coastal plain and mountainous area of the state. It is the best-conserved forest area compared to the other sites, has a sloping relief, and a warm (MAT 22.4 °C) and wet (MAP 1539 mm) climate [17].

### 2.1. Entomological Surveys

Entomological surveys were conducted during and after the peak of the 2017 epidemic [16,17,18]. The collection of adult and immature mosquitoes was planned according to the behavior of traditional YFV vector species (i.e., *Haemagogus* and *Sabethes*), which have daytime habits and preferred breeding sites. Adult mosquitoes were captured with protected human attraction using nets and aspiration tubes [7,8,9,10,11,12,13,14,15,16,17,18,19].

Adult sampling 1: An exploratory survey was conducted during the peak of records of YFV infections in humans and NHPs. We investigated the diversity and distribution of mosquito species and characterized the local vectors. Mosquito collections were conducted at ground level from 8 February to 2 March 2017, between the 6th and 9th epidemiological weeks (EWs). We hypothesized that during this peak in YFV transmission, a higher proportion of competent vectors would have previously bitten viremic NHPs. At each sampling site, two to four collectors moved from the same departure point in opposite directions for 50–100 m, capturing mosquitoes for 30 min. The procedure was repeated for 3 to 8 h per sampling day and 2 to 5 d in each study area, covering a total area of approximately 4 ha.

Adult sampling 2: We investigated the vertical distribution of mosquitoes in the forest, as well as mosquito species diversity and infection rates of vectors. A monthly survey was conducted during the four months following the epidemic peak, from March (11th EW) to June (26th EWs) 2017, to investigate a distinct epidemiological scenario. Mosquito captures were conducted simultaneously at the ground and tree canopy levels once a month at VNa and PA. Two collectors worked for 3–8 h daily for two days at each study site. Canopy captures were performed on a platform at 15 m; ground-level captures were performed as described for adult sampling 1, with each collector sampling a total area of ~2 ha around the platform.

We estimated the biting frequency of mosquito species found naturally infected with YFV during both adult sampling times by dividing the total number of individuals belonging to the same species attempting to feed on one person by the total number of hours spent on capture by one person at each site.

Immature sampling: We investigated the abundance of the main vector species and the vertical transmission of YFV. After the epidemic peak, from 30 April to 3 June 2017 (between the 19th and 22nd EWs), traps for the collection of immature mosquitoes were installed in VNa and CA. We used traditional ovitraps, considered appropriate for collecting *Haemagogus* spp. [20], and bamboo traps (sections of bamboo internodes) with small lateral holes [21] simulating the natural larval habitats of several *Sabethes* spp. The ovitraps consisted of black plastic jars containing nearly 300 mL of spring water and one plywood paddle (Eucatex, São Paulo, Brazil) as support for oviposition [22]. We installed 40 traps per site; ten sets of traps were installed approximately 20 m apart. Each set was composed of four traps set in the same tree, with one ovitrap and one bamboo trap suspended at 6 m and another couple of traps at 1.2 m. The traps were collected 7 to 10 d after installation and refilled with fresh water without changing the paddle; the traps were operated for a similar period each month. The paddles were immersed in water to stimulate egg hatching in the laboratory. The immature forms were reared until adult emergence, according to Consoli and Lourenço-de-Oliveira (1994) [19].

Adult mosquitoes captured during fieldwork or reared in the laboratory were immediately frozen in liquid nitrogen for investigation of viral infection.

### 2.2. Taxonomic Characterization

Species identification was performed on a cold table using the taxonomic keys [19,23,24,25,26]. Voucher specimens were deposited in the Culicidae Collection, Instituto Oswaldo Cruz. Mosquitoes were pooled by species, location, and sampling date in an L15 culture medium with 20% fetal bovine serum. A pool contained a maximum of five individuals if they belong to species of tribe Aedini (genera *Haemagogus, Aedes,* and *Psorophora*) and genus *Sabethes*, that include those considered primary or secondary vector, or ten individuals if the genus was not yet recognized as a YFV vector. So, after all mosquitoes of a given species, site, and sampling time were grouped according to this criterion, the remaining mosquitoes composed conspecific pools with a smaller number of individuals. Blood-fed female and male specimens were stored in separate cryotubes. Samples were stored at −80 °C for viral genome detection.

### 2.3. YFV Detection

Mosquito pools were ground in a Precellys 24 tissue homogenizer (Bertin Corp, Rockville, MA, USA) and centrifuged at 9600× *g* for 10 min at 4 °C. RNA was extracted from 140 µL of the supernatant using a QIAamp Viral RNA Mini Kit (Qiagen, Germantown, MA, USA) according to the manufacturer’s instructions. YFV detection was performed by RT-qPCR using a “Yellow Fever IBMP Kit” (1-step and multiplex with internal control)—produced by the Institute of Molecular Biology of Paraná—and QuantStudio 6 Flex Real-Time PCR System (Applied Biosystems, Foster City, CA, USA). The samples were examined in duplicate; negative and positive controls were used for each reaction as previously described [7].

### 2.4. Statistical Analysis

Data were analyzed using R software [27]. Mosquito community composition at each sampling site and sampling time was determined with the Shannon-Weaver Diversity Index (H’) [28]. The frequency of each mosquito species in the vertical extracts of the forest (ground and canopy levels) based on the adult sampling 2 data were compared using a non-parametric Mann–Whitney U test with α < 0.05. Infection rates were calculated using the R package PooledInfRate. The minimum infection rate (MIR) was obtained by dividing the number of YFV-positive pools by the total number of adults for the species multiplied by 1000. The maximum likelihood estimate per 1000 mosquitoes (MLE) was calculated as = 1 − (1 − Y/X) ^ (1/m), where Y is the number of positive pools, X is the total number of pools, and m is the size of each tested pool. Graphs were produced using the R package ggplot2 [29]. Maps were created using QGIS 3.12.2 [30].

## 3. Results

### 3.1. Entomological Survey Results

A total of 10,658 mosquitoes from 14 genera and 78 species were collected (Table 1 and Table S1), of which 99.87% and 0.13% were Culicinae and Anophelinae, respectively. Most samples were adult mosquitoes (95.17%), and 515 immature individuals (4.83%) were collected in ovitraps (Table 1).

The Sabethini tribe was the most abundant (80.67%) and had the highest species richness (51 taxa), followed by the Aedini tribe (16.62% with 14 species, Table 1).

The ten most common species were, in decreasing order of abundance, *Wyeomyia palmata*/*galvaoi* (10.69%), *Wy.* aff. *davisi* (9.90%), *Limatus durhamii* (9.49%), *Wy. incaudata* (5.11%), *Li. pseudomethysticus* (4.64%), *Haemagogus leucocelaenus* (4.45%), *Wy. edwardsi* (3.90%), *Ae. scapularis* (3.81%), *Trichoprosopon castroi*/*similis* (3.64%), and *Shannoniana fluviatilis* (3.13%).

Mosquito species diversity (Shannon-Weaver index) varied among sampling sites and sampling time, with STb displaying the lowest H’ values and CA and PA the highest (Table 1 and Table S2). *Hg*. *leucocelaenus*, *Psorophora ferox*, *Onirion personatum*, *Wy. lutzi*, *Wy. sabethea*, *Wy. bourrouli*/*forcipenis* and species of the Serratus group of *Aedes* [19,31] were found in all six sites.

At least one of the primary YFV vectors in southeast Brazil—*Hg. leucocelaenus* or *Hg. janthinomys/capricornii* (0.66% abundance)—were detected at all six sites. *Hg. janthinomys/capricornii* was not captured at VNb but was present in all four municipalities included in the study (Supplementary Table S2). Species of Sabethini, which are considered to play a role in YFV transmission, were heterogeneously distributed: *Sabethes chloropterus* was found in PA and STa (abundance of 0.35%); *Sa. soperi* was captured at CA and STa (1.62%); and *Sa. albiprivus* (0.48%) was found in all municipalities but was absent at two sites: STb and VNb. Species of the tribe Aedini—potential vectors or naturally infected by YFV—were captured at all six study sites, including *Ae. serratus* (group) (0.90%), *Ps. ferox* (2.51%), *Ae. scapularis* (3.81%, absent only in VNb), and *Ps. albipes* (0.69%, found only in PA). *Ae. albopictus* (2.00%) was found at all sites except VNa. One larva of *Ae. aegypti* was collected at STa (Supplementary Table S2).

The YFV vectors *Hg. leucocelaenus*, *Hg. janthinomys/capricornii*, and *Sa. chloropterus*—were found in both adult sampling procedures but not in immature sampling (Table 1 and Table S2). Among them, *Hg. leucocelaenus* was the most abundant in the adult samplings. The average bite frequencies by YFV vectors were 3.33 and 1.42 bites per person/h in adult sampling 1 and 2, respectively (Table 2). The highest bite frequencies were recorded in CA (6.47), STb (3.23), and PA (2.81), all during adult sampling 1.

The most abundant mosquito species at each study site during adult sampling 1 are shown in Figure 3. Unlike at the other study sites where Sabethini mosquitoes prevailed, Aedini mosquitoes were among the most abundant in ST (STa and STb), and also where the traditional primary YFV vectors were most abundant, totaling 13.23% of the mosquitoes collected.

**Figure 4:**
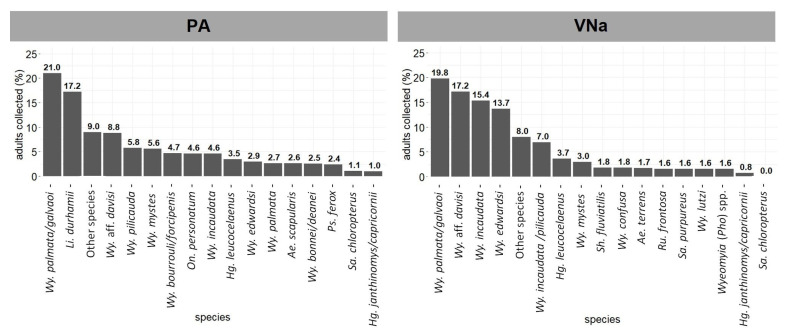
Most common mosquito species in adult sampling 2 in Pancas (PA) and Venda Nova do Imigrante (VNa), Espírito Santo, from March to June 2017.

Although the species richness and diversity (Shannon-Weaver diversity index) at PA and VNa changed only slightly between adult sampling 1 and 2, the dominant species varied markedly (Supplementary Table S2). *Wy. palmata/galvaoi* was the most abundant species at both sites during adult sampling 2 (Figure 4): 21.01% in PA and 19.77% in VNa. Other species of the subgenus *Phoniomyia*, including *Wy*. aff. *davisi*, *Wy. pilicauda, Wy*. *incaudata*, and *Wy. edwardsi* were also comparatively abundant at both sites. In PA, *Hg. leucocelaenus*, *Sa. chloropterus*, and *Hg. janthinomys/capricornii* comprised 3.46%, 1.08%, and 0.98% of the species, respectively. In VNa, *Hg. leucocelaenus* accounted for 3.66% of the species (Figure 4), whereas *Hg. janthinomys/capricornii* accounted for only 0.75%.

*Li. durhamii*, *Wy. mystes*, *Wy. bourrouli/forcipenis*, and *Ae. scapularis* were significantly more abundant at ground level (Supplementary Table S3). In contrast, *Hg. janthinomys/capricornii* and *Wy. edwardsi* prevailed in forest canopies. There was no significant difference in abundance between canopy and ground collections for the other species whose total collected allowed the analysis.

**Figure 5:**
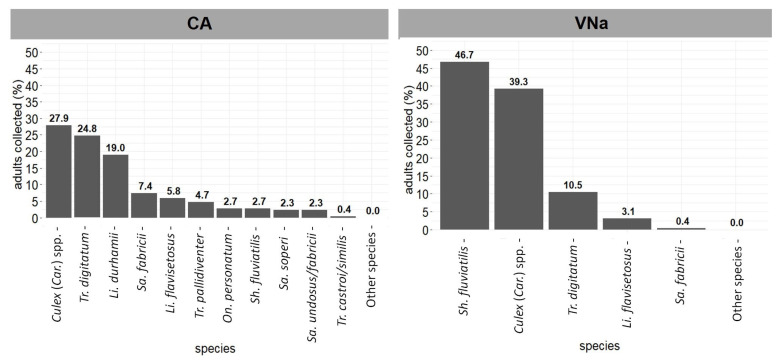
Mosquito species (in decreasing order of abundance) found in immature sampling in Cariacica (CA) and Venda Nova do Imigrante (VNa), Espírito Santo, from April to June 2017.

In immature samples, *Culex* (Car.) spp. (27.91%), *Tr. digitatum* (24.81%), and *Li. durhamii* (18.99%) were the most abundant in CA, whereas *Sh. fluviatilis* (46.69%), *Culex* (Car.) spp. (39.30%), and *Tr. digitatum* (10.51%) were the most abundant in VNa (Figure 5).

### 3.2. Detection of Natural Infection by YFV

We tested a total of 963 pools comprising 6712 mosquitoes. All Aedini and *Sabethes* were tested, as well as the most abundant species belonging to other genera of Sabethini were collected during the peak transmission (Supplementary Table S4).

Infections were detected at all study sites during adult sampling 1. In contrast, no positive sample was detected in adult sampling 2 and immature sampling.

We detected 19 YFV-positive pools of seven species. Considering the total collected in the six sampling sites, their MIR in descending order were: *Sa. chloropterus* (MIR = 62.5, MLE = 43.6), *Hg. janthinomys/capricornii* (MIR = 54.1, MLE = 35.8), *Sa. identicus* (MIR = 38.5, MLE = 26.4), *Hg. leucocelaenus* (MIR = 32.5, MLE = 32.1), *Ae. aureolineatus* (MIR = 14.3, MLE = 8.7), *Sa. soperi* (MIR = 12.3, MLE = 12.1), and *Sh. fluviatilis* (MIR = 5.9, MLE = 5.4) (Supplementary Table S4).

Infections in *Hg. leucocelaenus* were recorded at all study sites except for PA, regardless of its high abundance. Moreover, *Hg. leucocelaenus* was the only species found to carry YFV in STb and VNa. In contrast, four species were found naturally infected with YFV in CA: *Hg. leucocelaenus*, *Hg. janthinomys/capricornii*, *Sa. identicus*, and *Sa. soperi*. Coincidently, this was where mosquito sampling started with the shortest time elapsed from the first YFV record in humans or NHPs (2 weeks, Supplementary Table S5). The two YFV-positive pools detected in PA comprised *Hg. janthinomys/capricornii* and *Sa. chloropterus*. *Ae. aureolineatus* was found to be positive for YFV only in STa (Supplementary Table S4). *Aedes aegypti* captured at STa tested negative, similar to potential or secondary YFV vectors, such as *Ae. albopictus*, *Psorophora* spp., and *Sa. albiprivus*.

The MIR and MLE values varied considerably according to mosquito species and site. The highest infection rates were observed in PA for *Hg. janthinomys/capricornii* (MIR and MLE = 500.00). This species displayed high MLE (129.45) in CA. The lowest infection rates for *Hg. leucocelaenus* were detected in STa (MIR = 13.51, MLE = 13.89), where one positive sample of *Ae. aureolineatus* (MIR = 71.43, MLE = 117.75) was recorded (Table 3). In contrast, the highest MIR and MLE values were observed for *Hg. leucocelaenus* at the two sites of VN (VNa and VNb). There was no linear correlation (Pearson correlation coefficient; *ρ* = −0.04) between MIRs of *Haemagogus* species and the Shannon-Weaver index (H’). The sample size and study design prevented any statistical analysis from comparing mosquito species diversity and MIRs with human or monkey YFV infections in municipalities surveyed during and after the peak of transmission.

## 4. Discussion

This study presents the most comprehensive investigation of sylvatic mosquito fauna in terms of the number of study sites and sampling efforts in ES. Mosquito surveys were carried out during the 2017–2019 YFV outbreak in southeast Brazil [7,10,32,33,34]; however, to the best of our knowledge, this is the only study of a systematic vector collection from the beginning to the end of the transmission period in the same areas. Moreover, our results and previous data [7,35] demonstrate a great diversity of Culicidae species in ES, including several YFV vectors. Notably, our study included the same zone in ES where the first report of YFV transmission without *Ae. aegypti* was described [8].

When the sylvatic YFV outbreak was discovered in Canaan Valley, ST, in March 1932, the peak period of transmission had already passed. The investigations reported 83 suspected cases, with nine deaths from January to March 1932 [3,8]. Curiously, the peak of cases occurred during the same period (January and February) in the 2017 outbreak. A serological investigation made in the early 1930s revealed that 12% of people living near forests had antibodies against YFV and that undiagnosed YFV epizootic waves had crossed those rural areas via the forests in ES before. Canaan Valley was affected again in 1939, with 198 cases recorded in ES [2,3]. These findings indicate that forest areas were considerably receptive to the transmission of YFV. Nevertheless, inexplicably, approximately 80 years passed before the 2017 outbreak. It is likely that unknown ecological changes that occurred during the recent decades facilitated the spread of the virus from the Cerrado biome in MG to this portion of the Atlantic Forest in ES.

In the 1930s, the most common mosquito species found in ST was *Ae. scapularis*, followed by other Aedini species, such as *Psorophora* spp., *Ae. serratus*, and *Ae. terrens.* In the present study, *Ae. scapularis* was the most abundant mosquito found in STa and the third-most abundant in STb. *Psorophora* spp., *Ae. serratus*, and *Ae. terrens* maintained high abundances in 2017 (Figure 3). Intriguingly, *Haemagogus* spp., which was common and widespread in the 2017 samples, was not even recorded in the 1930s. Entomological surveys carried out during the 1932 outbreak could not determine the vectors, and other hematophagous insects besides mosquitoes, such as sand flies, were also suspected [3,8]. The role of *Ae. scapularis* as a secondary YFV vector was much later suggested in other Brazilian areas [5,7,36]. The primary role of *Hg. leucocelaenus* and *Hg. capricornii* as vectors and the potential of undetermined Sabethini species in YFV transmission were first described in the Paraíba do Sul River basin in RJ [3,37]. Thus, the YFV vectors in ES, particularly where sylvatic yellow fever was first described, remain to be elucidated.

Mosquito species composition is affected by climate, environmental conditions, and landscape topography [20,33,38,39]. Therefore, we surveyed six YFV foci located at altitudes ranging from 170 m to 1140 m in three river basins (Itapemirim, Doce, and Coastal), representing distinct vegetation structures, rainfall, and temperature.

When data on biting frequency, abundance, and YFV infection rates in 2017 are considered, the role of *Hg. leucocelaenus* and *Hg. janthinomys/capricornii* as primary vectors becomes evident. Although they presented distinct biting frequencies, *Hg. leucocelaenus* was detected in all study sites and was the most abundant vector species in four of them. One person was exposed to approximately two bites of *Hg. leucocelaenus* per hour during the daytime in all areas except for the two high-altitude VN sites. However, low biting frequencies in the VN sites were compensated for by high infection rates, that is, half or more of *Hg. leucocelaenus* pools were positive for the YFV (Table 3 and Table S4). These features reinforce its role as a primary vector, as vectorial capacity is influenced by abundance, biting frequencies, and infection rates.

*Hg. leucocelaenus* is distributed throughout Brazil and has been recorded in several municipalities of ES [7,19,35,40]. In this study, it was detected at all study sites at both the tree canopy and ground levels, consistent with previous results [41,42]. The vertical movement of *Hg. leucocelaenus* inside and near the epizootic forest would favor the transmission of YFV from infected NHPs to humans.

*Hg. janthinomys/capricornii* was significantly more abundant in the tree canopy, as expected [43,44]. This species has been recognized for decades as the primary vector of YFV in South America, especially in the Amazon, where it is much more abundant than *Hg. leucocelaenus* [37,44,45,46,47]. Outside of the Amazon, such as in Rio Grande do Sul (where important YFV outbreaks occurred in 2003, 2008–2009, and 2020–2021), *Hg. janthinomys/capricornii* does not occur, and *Hg. leucocelaenus* has been s considered to be the primary vector [6,48]. *Hg. janthinomys/capricornii* exhibited high infection rates in two sites. *Hg. leucocelaenus* had high abundance and biting rates and was detected in five of the six study sites. For instance, in PA *Hg. leucocelaenus* was quite frequent but was not found infected, whereas one of the two captured *Hg. janthinomys/capricornii* were detected infected during the transmission peak. Therefore, the co-occurrence of *Hg. janthinomys/capricornii* and *Hg. leucocelaenus* was certainly a key factor promoting YFV transmission in the 2017 outbreak in ES, as well as elsewhere in Southeast Brazil in 2017–2019 [7,10,32].

YFV infections were detected in five other mosquito species, including *Sa. identicus*, *Ae. aureolineatus*, *Sh. fluviatilis*, *Sa. soperi*, and *Sa. chloropterus*. However, all of these species were detected at sites where the primary vectors (*Haemagogus*) were also found to harbor the virus. In contrast to *Hg. leucocelaenus* and *Hg. janthinomys/capricornii*, these five species had a more limited distribution, exhibited lower abundance and bite frequency, and natural infection was recorded in only one of the six sites with usually low MIR and MLE. These features suggest a secondary, local, or momentary role in transmission for these species. This was the case with *Sa. identicus*, *Ae. aureolineatus*, and *Sh. fluviatilis.* Their natural infection with YFV was detected for the first time in this study. *Sa. soperi* was once detected infected in the Cerrado [46,49], and *Sa. chloropterus* was recorded as infected several times across the Americas, including the Atlantic Forest [7,45,46,50]. *Sh. fluviatilis* and any other *Shannoniana* species have never been associated with any arbovirus transmission cycle [46,49]. Although *Ae. aureolineatus* and *Sa. identicus* belong to genera commonly involved in YFV transmission, the vector competence for YFV of these two species and *Sh. fluviatilis* has never been accessed. From our analysis, we do not know whether these species are capable of transmitting the virus or if the infection is restricted to the primary tissue of viral replication (the stomach) because the pools include the entire mosquito body. In any case, ecological and environmental conditions may explain the detection of YFV infections in a greater diversity of mosquito species. The YFV epizootic wave of 2017 quickly infected thousands of naïve NHPs. Dying or very sick viremic NHPs usually tend to descend to low forest strata or lie lethargic on the ground, where they provide mosquitoes of several species with an ideal opportunity for infection [51]. This scenario could likely occur at the CA site with the largest and most preserved fragment of the Atlantic rain forest sampled here, which exhibited great richness (57 taxa) and high levels of natural infection in *Hg. leucocelaenus*, *Hg. janthinomys/capricornii*, *Sa. soperi*, and *Sa. identicus*.

In January 2017, the YFV spread from Minas Gerais through the Rio Doce and Rio Itapemirim basins and occupied a large part of the forest fragments in only a few weeks [11]. A large number of human and NHP cases [9,18], as well as the rapid spread across ES (Figure 1), illustrated the high sensitivity of this area at the time. This was probably due to the abundance of both competent primary vectors and susceptible NHPs under favorable environmental and climatic conditions (during the rainy season).

The rapid spread of the epizootic wave leading to the death or immunization of NHPs also helps explain the short period of virus circulation in ES. Records of YFV circulation, whether in humans or NHPs, were recorded at all study sites until the first half of March 2017 (Figure 2). Due to this pattern, epizootic YFV waves in the extra-Amazon area have been compared to “fires” that consume themselves and rarely return or remain in recently affected areas, in which future epizootics will depend on viral reintroduction from a new wave initiated in the Amazon, the endemic area. [2,11,51,52]. This could explain why the YFV-positive mosquito pools were detected only at adult sampling 1 (during the 6th–9th EWs), at the peak of YFV transmission. No YFV-positive mosquito was recorded during adult sampling 2 (March to June 2017), even in the forest canopy—the preferred habitat of the primatophilic primary vectors. The absence of the YFV in mosquitoes that emerged from ovitraps (operated from April–June 2017) and in the adult sampling 2 further confirms that virus transmission occurred at a low frequency or had apparently been interrupted. It is important to note that none of the immature mosquitoes collected were primary vectors of YFV, and only one secondary vector (*Sa. soperi*) was collected.

Phylogeographic analyses of YFV transmission in Southeast Brazil suggested an average velocity of 0.12 km/d [53] and 0.5 km/d [11]. The arrival and peak of human cases and NHP deaths in ES occurred in the summer of 2017 (January to early March). Epizootics were first recorded in Ibatiba, western ES, in the 1st EW of 2017 (Figure 1). Epizootics were first detected in CA, approximately 120 km east of the state, in the 7th EW. This epidemiological data suggest that the speed of YFV transmission in the Atlantic Forest of ES was ~2.9 km/d, similar to the 2.7 km/d obtained with an epidemiological model assessing NHP deaths during the warmer months of the 2017 outbreak in SP [54] and the 3.3 km/d obtained in a phylogeographic analysis of 2017–2018 viral samples from southeast Brazil [55].

Our findings emphasize the need for rapid entomo-virological investigation in case of suspected YFV infections. For example, in PA, where it took six weeks from the first sign of YFV circulation to the beginning of mosquito collections (Supplementary Table S5), no YFV-positive sample was detected in *Hg. leucocelaenus*, even though it was abundant at this site. (Table 2 and Table 3). On the other hand, several positive samples of *Hg. leucocelaenus* were found at other sites between the 2nd and 5th weeks after the first suspicion of viral circulation (Table 3 and Table S5). Moreover, the highest number of positive pools and YFV-positive species were recorded two weeks after the first YFV case in CA. It has been suggested that there is a greater chance of collecting infected mosquitoes up to 24 d after the first signs of transmission [7] or 41 d after the last detected YFV case [46]. Thus, the earlier entomological investigations are initiated following the first suspicion of YFV (death of NHPs), the greater the chance of detecting infected mosquitoes and elucidating transmission dynamics, especially in terms of the diversity of mosquitoes involved in viral circulation.

By systematically sampling and screening for natural infections in geographically distinct and topographically heterogeneous localities, we generated an annotated checklist of mosquito species in the state of ES, which can be applied in future studies of arboviruses and other mosquito-related parasites. It also provided an opportunity to train local stakeholders in medical entomology, which could aid in restoring large-scale YFV surveillance in ES and be replicated for future outbreaks. Our study showed that ES remains a highly sensitive area for the circulation of YFV, as the main vector species are abundant and widespread among small- or medium-sized forest fragments to large, well-preserved Atlantic Forest patches at 170 to 1140 m in altitude with distinct climatic regimes. These findings reinforce the importance of maintaining high vaccination coverage in these historically affected areas, in line with the recent Pan American Health Organization guidelines [56]. In this way, mosquito surveillance, combined with the use of new methodologies, such as the modeling of risk areas and applications for monitoring epizootic diseases [51,57], can contribute to improving the surveillance and control of the sylvatic YFV.

## 5. Conclusions

After extensive sampling, taxonomic identification, and molecular diagnostic efforts, we concluded that *Haemagogus leucocelaenus* and *Hg. janthinomys/capricornii* were the main YFV vectors during the 2017 outbreak due to their higher abundance and YFV infection rates in ES areas. Moreover, other vectors may have a secondary role in transmission, such as *Sabethes chloropterus*, *Sa. soperi*, *Sa. identicus*, *Aedes aureolineatus*, and *Shannoniana fluviatilis,* as they were also found naturally infected. The study emphasizes the importance of monitoring mosquito communities in the Atlantic Forest and maintenance of high vaccination coverage in receptive areas to YFV transmission.

## Figures and Tables

**Table 1:** Mosquito species identified at the six study sites located in four municipalities of Espírito Santo, including Cariacica (CA), Pancas (PA), Venda Nova do Imigrante (VNa and VNb), and Santa Teresa (STa and STb), from February to June 2017.

Taxon	Adults						Immature		Total	Ab (%) ^1^
	CA	PA	STa	STb	VNa	VNb	CA	VNA		
Subfamily Anophelinae	2		4		8				14	0.1
Tribe Anophelini	2		4		8				14	0.1
*Anopheles* (*Kerteszia*) *cruzii* Dyar & Knab, 1908			3		4				7	0.07
*Anopheles (Kerteszia) laneanus?* (Corrêa & Cerqueira, 1944)					2				2	0.02
*Anopheles* (*Kerteszia*) sp.					2				2	0.02
*Anopheles* (*Nyssorhynchus*) *triannulatus* (Neiva & Pinto, 1922)			1						1	0.01
*Anopheles* (*Stethomyia*) *nimbus* (Theobald, 1902)*/thomasi* Shannon, 1933*/acanthotorynus* Komp 1937	2								2	0.02
Subfamily Culicinae	2005	3170	886	310	2677	1081	258	257	10,644	99.9
Tribe Aedini	181	749	439	184	181	37			1771	16.6
*Aedes* (*Howardina*) *aureolineatus* Berlin, 1969	35		17	1	29				82	0.77
*Aedes* (*Howardina*) *fulvithorax* (Lutz, 1904)	2				2				4	0.04
*Aedes* (*Ochlerotatus*) *scapularis* (Rondani, 1848)	2	183	187	31	3				406	3.81
*Aedes* (*Ochlerotatus*) *serratus* (Theobald, 1901)/*hastatus Dyar, 1922*/*oligopistus Dyar, 1918*/*eucephalaeus Dyar, 1918*	2	52	38	1	2	1			96	0.90
*Aedes* (*Ochlerotatus*) *taeniorhynchus* (Wiedemann, 1821)			1						1	0.01
*Aedes* (*Protomacleaya*) *terrens* (Walker, 1856)	3	3	16		43	3			68	0.64
*Aedes* (*Stegomyia*) *aegypti* (Linnaeus,1762)			1						1	0.01
*Aedes* (*Stegomyia*) *albopictus* (Skuse, 1894)	7	95	4	106		1			213	2.00
*Aedes* sp.			1			1			2	0.02
*Haemagogus* (*Haemagogus*) *janthinomys* Dyar, 1921/*Hg. capricornii* Lutz, 1904	10	21	7	17	15				70	0.66
*Haemagogus* (*Conopostegus*) *leucocelaenus* (Dyar & Shannon, 1924)	112	171	74	24	86	7			474	4.45
*Psorophora* (*Janthinosoma*) *albigenu* (Peryassú, 1908)		1							1	0.01
*Psorophora* (*Janthinosoma*) *albipes* (Theobald, 1907)		74							74	0.69
*Psorophora* (*Janthinosoma*) *albipes* (Theobald, 1907)/*albigenu* (Peryassú, 1908)/*varipes* (*Coquillett, 1904*)		3							3	0.03
*Psorophora* (*Janthinosoma*) *ferox* (von Humboldt, 1819)	8	141	91	4	1	22			267	2.51
*Psorophora* (*Janthinosoma*) *lanei* Shannon and Cerqueira, 1943		1	1			2			4	0.04
*Psorophora* (*Janthinosoma*) sp.		4	1						5	0.05
Tribe Culicini	8	26	20		22	12	72	101	261	2.4
*Culex* (*Carrollia*) sp.							72	101	173	1.62
*Culex* (*Culex*) Declarator Group			2						2	0.02
*Culex* (*Culex*) *declarator* Dyar and Knab, 1906		1							1	0.01
*Culex* (*Culex*) *nigripalpus* Theobald, 1901	3	6	9		2				20	0.19
*Culex* (*Culex*) sp.	2	6			7	2			17	0.16
*Culex* (*Melanoconion*) sp.					9	7			16	0.15
*Culex* (*Microculex*) *neglectus* Lutz, 1904		1			1				2	0.02
*Culex* (*Microculex*) *imitator* Theobald, 1903		1							1	0.01
*Culex* (*Microculex*) sp.						2			2	0.02
*Culex* sp.	3	11	9		3	1			27	0.25
Tribe Mansoninii	1	6	1	6					14	0.1
*Coquillettidia* (*Rhynchotaenia*) *albicosta* (Peryassú, 1908)	1								1	0.01
*Coquillettidia* (*Rhynchotaenia*) *chrysonotum* (Peryassú, 1922)			1						1	0.01
*Mansonia* (*Mansonia*) *titillans* (Walker, 1848)		5		6					11	0.10
*Mansonia* (*Mansonia*) *sp.*		1							1	0.01
Tribe Sabethini	1815	2389	426	120	2474	1032	186	156	8598	80.7
*Limatus durhamii* Theobald, 1901	264	646	31	7	14		49		1011	9.49
*Limatus flavisetosus* Oliveira Castro, 1935	18	1	1		5		15	8	48	0.45
*Limatus pseudomethysticus* (Bonne-Wepster & Bonne, 1920)	329		134	4	28				495	4.64
*Limatus flavisetosus*? Oliveira Castro, 1935	1								1	0.01
*Limatus* sp.					1				1	0.01
*Onirion personatum* (Lutz, 1904)	34	93	17	60	31	36	7		278	2.61
*Runchomyia* (*Runchomyia*) *cerqueirai* (Stone, 1944)	25		1		3				29	0.27
*Runchomyia* (*Runchomyia*) *frontosa* (Theobald, 1903)	26	7	47		93	1			174	1.63
*Runchomyia* (*Runchomyia*) *humboldti* (Lane & Cerqueira, 1942)	28		5		3				36	0.34
*Runchomyia* (*Runchomyia*) *reversa* (Lane & Cerqueira, 1942)	1								1	0.01
*Runchomyia* (*Runchomyia*) *theobaldi*	2								2	0.02
*Runchomyia* (*Runchomyia*) sp.		1				1			2	0.02
*Sabethes* (*Davismyia*) *petrocchiae* (Shannon & Del Ponte, 1928)		10		22	4				36	0.34
*Sabethes* (*Peytonulus*) *aurescens* (Lutz, 1905)	21				6				27	0.25
*Sabethes* (*Peytonulus*) *fabricii* Lane & Cerqueira, 1942							19	1	20	0.19
*Sabethes* (*Peytonulus*) *undosus* (Coquillett, 1906)/*fabricii* Lane & Cerqueira 1942	90		3		1		6		100	0.94
*Sabethes* (*Peytonulus*) *hadrognathus Harbach, 1995*	1								1	0.01
*Sabethes* (*Peytonulus*) *identicus* Dyar & Knab, 1907	19	1			3	5			28	0.26
*Sabethes* (*Peytonulus*) aff. *Ignotus*	7				1				8	0.08
*Sabethes* (*Peytonulus*) *soperi* Lane & Cerqueira, 1942	166		1				6		173	1.62
*Sabethes* (*Peytonulus*) *whitmani* Lane and Cerqueira, 1942	3								3	0.03
*Sabethes* (*Peytonulus*) sp.	1								1	0.01
*Sabethes* (*Sabethes*) *albiprivus* Theobald, 1903	3	7	11		30				51	0.48
*Sabethes* (*Sabethes*) *batesi* Lane & Cerqueira, 1942	1	1							2	0.02
*Sabethes* (*Sabethes*) *forattinii* Cerqueira, 1961		10							10	0.09
*Sabethes* (*Sabethes*) *purpureus* (Theobald, 1907)	3				32	3			38	0.36
*Sabethes* (*Sabethinus*) *intermedius* (Lutz, 1904)	2		1						3	0.03
*Sabethes* (*Sabethinus*) *melanonymphe* Dyar, 1924	3	1							4	0.04
*Sabethes* (*Sabethinus*) *xhyphydes* Harbach, 1994	1	1	1						3	0.03
*Sabethes* (*Sabethoides*) *chloropterus* (von Humboldt, 1819)		33	4						37	0.35
*Sabethes* sp.	18								18	0.17
*Shannoniana* (*Shannoniana*) *fluviatilis* (Theobald, 1903)	46		11		114	36	7	120	334	3.13
*Trichoprosopon castroi* Lane & Cerqueira, 1942/*similis* Lane & Cerqueira, 1942	226		19	6	123	13	1		388	3.64
*Trichoprosopon compressum* Lutz, 1905	6	1			2	5			14	0.13
*Trichoprosopon digitatum* Rondani, 1848	6						64	27	97	0.91
*Trichoprosopon pallidiventer* (Lutz,1905)	35	1	35		52	40	12		175	1.64
*Trichoprosopon soaresi* Lane & Cerqueira, 1942	6					2			8	0.08
*Trichoprosopon* sp.	1				1				2	0.02
*Wyeomyia* (*Cruzmyia*) *dyari* Lane & Cerqueira, 1942	36				4				40	0.38
*Wyeomyia* (*Miamyia*) *codiocampa* Dyar & Knab, 1907			1		1	5			7	0.07
*Wyeomyia* (*Miamyia*) *limai*	2				1				3	0.03
*Wyeomyia* (*Miamyia*) *lutzi* (Costa Lima, 1930)	8	1	12	3	48	15			87	0.82
*Wyeomyia* (*Miamyia*) *oblita* (Lutz, 1904)		15							15	0.14
*Wyeomyia* (*Miamyia*) *sabethea* Lane & cerqueira, 1942	10	11	5	1	2	4			33	0.31
*Wyeomyia* (*Phoniomyia*) *antunesi* (Lane & Guimarães, 1937)	6								6	0.06
*Wyeomyia* (*Phoniomyia*) *bonnei* (Lane and Cerqueira, 1942)/*deanei* (Lourenço-de-Oliveira, 1983)	6	75			10	43			134	1.26
*Wyeomyia* (*Phoniomyia*) aff. *davisi*	33	212	17		391	402			1055	9.90
*Wyeomyia* (*Phoniomyia*) *edwardsi* (Lane & Cerqueira, 1942)	8	64	19		306	19			416	3.90
*Wyeomyia* (*Phoniomyia*) *incaudata* (Root, 1928)	78	117	16		301	33			545	5.11
*Wyeomyia* (*Phoniomyia*) *pilicauda* Root, 1928	20	131			10	10			171	1.60
*Wyeomyia* (*Phoniomyia*) *incaudata* (Root, 1928)/*pilicauda* Root, 1928					154	1			155	1.45
*Wyeomyia* (*Phoniomyia*) *muehlensi* Petrocchi, 1927	2	39			24	19			84	0.79
*Wyeomyia* (*Phoniomyia*) *palmata* (Lane & Cerqueira, 1942)		53							53	0.50
*Wyeomyia* (*Phoniomyia*) *palmata* (Lane & Cerqueira, 1942)/*galvaoi* (Correa & Ramalho, 1956)	20	454	12		408	245			1139	10.69
*Wyeomyia* (*Phoniomyia*) *theobaldi* (Lane & Cerqueira, 1942)		2	3		9	4			18	0.17
*Wyeomyia* (*Phoniomyia*) sp.	8	34	8		52	48			150	1.41
*Wyeomyia* (*Prosopolepis*) *confusa* (Lutz, 1905)	1	1			108	40			150	1.41
*Wyeomyia* (*Spilonympha*) *bourrouli* (Lutz, 1905)/*forcipenis* Lourenço-de-Oliveira & Silva, 1985	46	212	7	15	8	1			289	2.71
*Wyeomyia* (*Spilonympha*) *mystes* Dyar, 1924	73	135	3	2	84				297	2.79
*Wyeomyia* (*Triamyia*) *aporonoma Dyar and Knab 1906*	38								38	0.36
*Wyeomyia* (*Triamyia*) *aporonoma Dyar and Knab 1906/staminifera* Lourenço-de-Oliveira, Motta & Castro,1992	12								12	0.11
*Wyeomyia shannoni* Lane & Cerqueira, 1942	8								8	0.08
*Wyeomyia* (*Wyeomyia*) sp.		1							1	0.01
*Wyeomyia* sp.	7	18	1		6	1			33	0.31
Total	2007	3170	890	310	2685	1081	258	257	10,658	-
	10,143						515			
N. Taxa ^2^	57	44	42	17	47	29	11	5		

^1^ Ab (%) = Relative abundance calculated by dividing the number of mosquitoes of one species by the number of mosquitoes of all species × 100. ^2^ Number of taxa sampled at each site.

**Table 2:** Biting frequency of YFV vector species in Cariacica (CA), Pancas (PA), Venda Nova do Imigrante (VNa and VNb), and Santa Teresa (STa and STb), Espírito Santo, from February to June 2017.

	Adults Sampling ^1^														Adults Sampling ^2^					
	CA		PA		STa		STb		VNa		VNb		Total		PA		VNa		Total	
Sampling Effort ^1^ (h)	60		42		50.5		13		52		33		250.5		97		66		163	
Species	N ^2^	BF ^3^	N	BF	N	BF	N	BF	N	BF	N	BF	N	BF	N	BF	N	BF	N	BF
*Hg*. *leucocelaenus*	112	1.87	104	2.48	74	1.47	24	1.85	18	0.35	7	0.21	339	1.35	67	0.69	68	1.03	135	0.83
*Hg. janthinomys*/*capricornii*	10	0.17	2	0.05	7	0.14	17	1.31	1	0.02			37	0.15	19	0.20	14	0.21	33	0.20
*Sa*. *chloropterus*			12	0.29	4	0.08							16	0.06	21	0.22		0.00	21	0.13
*Ae. aureolineatus*	35	0.58			17	0.34	1	0.08	23	0.44			76	0.30			6	0.09	6	0.04
*Sa. identicus*	19	0.32							2	0.04	5	0.15	26	0.10	1	0.01	1	0.02	2	0.01
*Sa. soperi*	166	2.77			1	0.02							167	0.67					0	0.00
*Sh. fluviatilis*	46	0.77			11	0.22			80	1.54	36	1.09	173	0.69			34	0.52	34	0.21
TOTAL	388	6.47	118	2.81	114	2.26	42	3.23	124	2.38	48	1.45	834	3.33	108	1.11	123	1.86	231	1.42

^1^ Sampling effort = total number of sampling hours; ^2^ N = number of captured adults; ^3^ BF = Biting frequency, the number of mosquitoes attempting to feed on one person per hour.

**Table 3:** Rates of YFV natural infection in mosquitoes collected in adult sampling 1 at Cariacica (CA), Pancas (PA), Venda Nova do Imigrante (VNa and VNb), and Santa Teresa (STa and STb), Espírito Santo, 2017.

	Adults Sampling ^1^											
Local	CA		PA		STa		STb		VNa		VNb	
Species	MIR ^1^	MLE ^2^	MIR	MLE	MIR	MLE	MIR	MLE	MIR	MLE	MIR	MLE
*Ae. aureolineatus*	0.00	0.00			71.43	117.75	0.00	0.00	0.00	0.00		
*Hg. janthinomys/capricornii*	100.00	129.45	500.00	500.00	0.00	0.00	0.00	0.00	0.00	0.00		
*Hg. leucocelaenus*	36.04	38.96	0.00	0.00	13.51	13.89	83.33	102.19	166.67	338.45	142.86	276.98
*Sa. chloropterus*			83.33	91.44	0.00	0.00						
*Sa. identicus*	52.63	59.25							0.00	0.00	0.00	0.00
*Sa. soperi*	12.35	12.66			0.00	0.00						
*Sh. fluviatilis*	0.00	0.00			0.00	0.00			0.00	0.00	27.78	32.02

^1^ Minimum infection rate = the number of YFV-positive pools/number of adults tested for this species × 1000; ^2^ Maximum likelihood estimate per 1000 mosquitoes = 1 − (1 − Y/X) ^ (1/m), where Y is the number of positive pools, X is the total number of pools, and m is the size of each tested pool.

## Supplementary Materials

The following supporting information can be downloaded at: https://www.mdpi.com/article/10.3390/v14122805/s1, Table S1: Number of adults and immature mosquitoes collected during the entomological surveys from February to June 2017 in Cariacica (CA), Pancas (PA), Venda Nova do Imigrante (VNa and VNb), and Santa Teresa (STa and STb), Espírito Santo; Table S2: Number and percentage of adult mosquitoes belonging to the Aedini and Sabethini tribes (along with their potential role in yellow fever transmission^1^) collected in Cariacica (CA), Pancas (PA), Venda Nova do Imigrante (VNa and VNb), and Santa Teresa (STa and STb), Espírito Santo, before and after the peak of the epidemic (Adult sampling 1 and 2) from February to June 2017; Table S3: Mosquito species found at ground and canopy levels during adult sampling 2 in Pancas (PA) and Venda Nova do Imigrante (VNa), Espírito Santo, from March to June 2017; Table S4: Mosquito species tested for natural infection by the YFV in Cariacica (CA), Pancas (PA), Venda Nova do Imigrante (VNa and VNb), and Santa Teresa (STa and STb) from February to June 2017; Table S5: Number of YFV-positive mosquito pools correlated with the time elapsed between the first YFV record and the date of adult sampling 1 in Cariacica (CA), Pancas (PA), Venda Nova do Imigrante (VNa and VNb), and Santa Teresa (STa and STb), Espírito Santo, 2017.
