# Supplementary material for: Back to Where It Was First Described: Vectors of Sylvatic Yellow Fever Transmission in the 2017 Outbreak in Espírito Santo, Brazil
Source: Viruses. 2022 Dec 15;14(12):2805. doi: 10.3390/v14122805 (PMC9785321; doi:10.3390/v14122805)
Supplement: Supplementary file 1 [file viruses-14-02805-s001.zip › viruses-2069811-supplementary.pdf]

File: **Stanzani 2022_Viruses_Supplementary Material**

Manuscript titled:

Back to where it was first described: Vectors of sylvatic yellow fever transmission in the 2017 outbreak in Espírito Santo, Brazil

Corresponding authors:

Luciana Matos de Abreu Stanzani - luciana.abreu@ufes.br

Ricardo Lourenço-de-Oliveira - lourenco@ioc.fiocruz.br

Supplementary Table S1. Number of adults and immature mosquitoes collected per sampling method and time from February to June 2017 in Cariacica (CA), Pancas (PA), Venda Nova do Imigrante (VNa and VNb), and Santa Teresa (STa and STb), Espírito Santo.

Sampling	Sampling Method	Cronology	Level in the forest	Site	EW[1] when the 1st YFV was recorded	Number of mosquitoes collected per site	Total Number of collected mosquitoes
Adults 1	PHA[2]	February/ 2017 (6th - 9th EW)	Ground	VNa	3rd	829	6350
				VNb	3rd	1081	
				STa	3rd	890	
				STb	3rd	310	
				CA	7th	2007	
				PA	2nd	1233	
Adults 2	PHA	March to June/2017 (11th - 26th EW)	Ground and Canopy	VNa	3rd	1856	3793
				PA	2nd	1937	
Imma-ture	Ovitraps[3]	April to June/2017 (19th - 22nd)		VNa	3rd	257	515
				CA	7th	258	
Total							10658

[1]EW = Epidemiological week; [2]PHA = Protected Human Attraction; [3]Ovitraps = Traditional ovitraps and bamboo traps.

Supplementary Table S2. Adult mosquitoes belonging to the Aedini and Sabethini tribes with potential role in yellow fever transmission[1] collected in Cariacica (CA), Pancas (PA), Venda Nova do Imigrante (VNa and VNb), and Santa Teresa (STa and STb), Espírito Santo, during and after the peak of the epidemic (Adult samplings 1 and 2) from February to June 2017.

Species	Adult Sampling 1 (AS1)								Adult Sampling 2 (AS2)				AS1 + AS2 + Immature Sampling							
	CA	PA	STa	STb	VNa	VNb	Total	Ab (%)[3]	PA	VNa	Total	Ab (%)[3]	CA	PA	STa	STb	VNa	VNb	Total	Ab (%)[3]
Hg. leucocelaenus	112	104	74	24	18	7	339	5.34	67	68	135	3.56	112	171	74	24	86	7	474	4.45
Ae. scapularis	2	132	187	31	2		354	5.57	51	1	52	1.37	2	183	187	31	3		406	3.81
Sh. fluviatilis	46		11		80	36	173	2.72		34	34	0.90	53		11		234	36	334	3.13
Ps. ferox	8	95	91	4		22	220	3.46	46	1	47	1.24	8	141	91	4	1	22	267	2.51
Ae. albopictus	7	77	4	106		1	195	3.07	18		18	0.47	7	95	4	106		1	213	2.00
Sa. soperi	166		1				167	2.63				0.00	172		1				173	1.62
Ae. Serratus Group	2	31	38	1	2	1	75	1.18	21		21	0.55	2	52	38	1	2	1	96	0.90
Ae. aureolineatus	35		17	1	23		76	1.20		6	6	0.16	35		17	1	29		82	0.77
Ps. albipes		69					69	1.09	5		5	0.13		74					74	0.69
Hg. janthinomys/capricornii	10	2	7	17	1		37	0.58	19	14	33	0.87	10	21	7	17	15		70	0.66
Sa. albiprivus	3		11		17		31	0.49	7	13	20	0.53	3	7	11		30		51	0.48
Sa. chloropterus		12	4				16	0.25	21		21	0.55		33	4				37	0.35
Sa. identicus	19				2	5	26	0.41	1	1	2	0.05	19	1			3	5	28	0.26
Ae. aegypti			1				1	0.02				0.00			1				1	0.01
Sabethes spp.[4]	151	2	5	22	7	3	190	2.99	21	37	58	1.53	176	23	5	22	45	3	274	2.57
Subtotal	561	524	451	206	152	75	1969	31.00	277	175	452	11.91	599	801	451	206	448	75	2580	24.21
Other species	1446	709	439	104	677	1006	4381	68.99	1660	1681	3341	88.08	1666	2369	439	104	2494	1006	8078	75.79
Total	2007	1233	890	310	829	1081	6350		1937	1681	3793		2265	3170	890	310	2942	1081	10658	
Shannon-Weaver index	1.35	1.15	1.24	0.91	1.33	0.97	1.51		1.19	1.11	1.26		1.38	1.24	1.24	0.91	1.33	0.97	1.50	

[1]Mosquitoes species found naturally infected with YFV in previous studies and/or in this survey or demonstrated to be competent to transmit the virus experimentally. [2]Total of mosquitoes (adults and immatures) collected with all sampling methods and time. [3]Ab (%) = Relative abundance calculated by dividing the number of mosquitoes of one species by the number of mosquitoes of all species × 100. [4]Other species of genus *Sabethes*.

Supplementary Table S3. Mosquito species found at ground and canopy levels during adult sampling 2 in Pancas (PA) and Venda Nova do Imigrante (VNa), Espírito Santo, from March to June 2017.

		PA						VNa								
**Species**	**Level**	**March**	**April**	**May**	**June**	**Subtotal**		**March**	**April**	**May**	**June**	**Subtotal**		**Total**		**pValue[1]**
Wy. palmata/ galvaoi	Canopy	45	114	83	121	363	407	34	17	49	9	109	367	472	774	0.1275
	Ground	11	7	13	13	44		186	23	39	10	258		302		
Wy. aff. *davisi*	Canopy	14	50	31	63	158	171	61	24	42	15	142	319	300	490	0.0659
	Ground	6	1	3	3	13		112	21	39	5	177		190		
Wy. incaudata	Canopy	16	13	18	15	62	89		102	55	62	219	285	281	374	0.0651
	Ground	8	6	6	7	27		14	13	26	13	66		93		
Hg. leucocelaenus	Canopy	3	5	1	46	55	67	29	5	6		40	68	95	135	0.6698
	Ground	5			7	12		5	14	9		28		40		
On. personatum	Canopy	4	11	15	4	34	89				2	2	15	36	104	0.2875
	Ground	13	18	11	13	55		11		1	1	13		68		
Wy. edwardsi	Canopy	10	27	14	2	53	57	33	104	69	21	227	254	280	311	**0.0080**
	Ground	2	2			4			3	22	2	27		31		
Li. durhamii	Canopy	1				1	333					0	5	1	338	**0.0092**
	Ground	226	40	35	31	332			3		2	5		337		
Wy. mystes	Canopy	1	2			3	109		3		2	5	55	8	164	**0.0008**
	Ground	36	16	41	13	106		12	6	20	12	50		156		
Wy. pillicauda	Canopy	15	16	39	38	108	112		1	4		5	10	113	122	0.1470
	Ground	1	1	1	1	4			2	3		5		9		
Wy. incaudata/pillicauda	Canopy						0	129				129	129	129	129	0.3816
	Ground											0		0		
Wy. bourrouli/forcipenis	Canopy		3	4		7	91					0	4	7	95	**0.0433**
	Ground	45	13	14	12	84		1	3			4		88		
Ae. scapularis	Canopy						51					0	1	0	52	**0.0325**
	Ground	32	8		11	51					1	1		52		
Wy. bonnei/deanei	Canopy	8	13	16	7	44	49	1				1	5	45	54	0.1601
	Ground	4	1			5		4				4		9		
Ps. ferox	Canopy						**46**					**0**	**1**	**0**	**47**	0.0764
	Ground	44			2	**46**		1				**1**		**47**		

Wy. palmata	Canopy	11	4	6	29	**50**	**52**					**0**	**0**	**50**	**52**	0.0847
	Ground			2		**2**						**0**		**2**		
Hg. janthinomys/capricornii	Canopy	4	4		11	**19**	**19**	9	3	2		**14**	**14**	**33**	**33**	**0.0045**
	Ground											**0**		**0**		
Wy. muehlensi	Canopy						**38**					**0**	**0**	**0**	**38**	0.1709
	Ground	7	31			**38**						**0**		**38**		
Wyeomyia (*Pho.*) spp.	Canopy	3		10	3	**16**	**25**	1	1	1		**3**	**29**	**19**	**54**	0.2181
	Ground	2	4	2	1	**9**		15	1	2	8	**26**		**35**		
Ru. frontosa	Canopy						**5**	5				**5**	**30**	**5**	**35**	**N/A**
	Ground	3		2		**5**		11	2	6	6	**25**		**30**		
Sh. fluviatilis	Canopy						**0**					**0**	**34**	**0**	**34**	**N/A**
	Ground							26	3	2	3	**34**		**34**		
Wy. confusa	Canopy						**0**					**0**	**34**	**0**	**34**	**N/A**
	Ground							7	12	7	8	**34**		**34**		
Ae. terrens	Canopy						**1**	5	4	4		**13**	**32**	**13**	**33**	**N/A**
	Ground	1				**1**		5	5	8	1	**19**		**20**		
Sa. purpureus	Canopy						**0**	12	4	4	5	**25**	**29**	**25**	**29**	**N/A**
	Ground								1	1	2	**4**		**4**		
Wy. lutzi	Canopy						**0**	11	4		6	**21**	**29**	**21**	**29**	**N/A**
	Ground							2	2	2	2	**8**		**8**		
Ae. Serratus Group	Canopy						**21**					**0**	**0**	**0**	**21**	**N/A**
	Ground	19			2	**21**						**0**		**21**		
Sa. chloropterus	Canopy	4	7	6	1	**18**	**21**					**0**	**0**	**18**	**21**	**N/A**
	Ground	3				**3**						**0**		**3**		
Sa. albiprivus	Canopy		2	1	3	**6**	**7**	3	3	3		**9**	**13**	**15**	**20**	**N/A**
	Ground			1		**1**		1	2	1		**4**		**5**		
Tr. castroi/similis	Canopy						**0**					**0**	**19**	**0**	**19**	**N/A**
	Ground							16	3			**19**		**19**		
Ae. albopictus	Canopy		1			**1**	**18**					**0**	**0**	**1**	**18**	**N/A**
	Ground	14		1	2	**17**						**0**		**17**		
Li. pseudomethysticus	Canopy						**0**	2				**2**	**16**	**2**	**16**	**N/A**

	Ground							10	1		3	**14**		**14**		
Sa. petrocchiae	Canopy	1		1		**2**	**10**	1				**1**	**3**	**3**	**13**	**N/A**
	Ground	1	5	2		**8**				2		**2**		**10**		
Tr. pallidiventer	Canopy						**0**					**0**	**11**	**0**	**11**	**N/A**
	Ground							10			1	**11**		**11**		
Sa. forattinii	Canopy		1	5	4	**10**	**10**					**0**	**0**	**10**	**10**	**N/A**
	Ground											**0**		**0**		
Culex spp.	Canopy						**11**					**0**	**2**	**0**	**13**	**N/A**
	Ground	7		3	1	**11**			1		1	**2**		**13**		
Culex (*Cux.*) spp.	Canopy						**6**					**0**	**1**	**0**	**7**	**N/A**
	Ground	5	1			**6**				1		**1**		**7**		
Wyeomyia spp.	Canopy						**4**					**0**	**2**	**0**	**6**	**N/A**
	Ground	1	1	2		**4**		1			1	**2**		**6**		
Ma. titillans	Canopy						**5**					**0**	**0**	**0**	**5**	**N/A**
	Ground	5				**5**						**0**		**5**		
Ps. albipes	Canopy						**5**					**0**	**0**	**0**	**5**	**N/A**
	Ground	4	1			**5**						**0**		**5**		
Cx. neglectus	Canopy			1		**1**	**1**					**0**	**1**	**1**	**2**	**N/A**
	Ground										1	**1**		**1**		
Cx. nigripalpus	Canopy						**1**			1		**1**	**1**	**1**	**2**	**N/A**
	Ground			1		**1**						**0**		**1**		
Sa. identicus	Canopy						**1**	1				**1**	**1**	**1**	**2**	**N/A**
	Ground	1				**1**						**0**		**1**		
Cx. declarator	Canopy						**1**					**0**	**0**	**0**	**1**	**N/A**
	Ground			1		**1**						**0**		**1**		
Cx. imitator	Canopy			1		**1**	**1**					**0**	**0**	**1**	**1**	**N/A**
	Ground											**0**		**0**		
Mansonia (*Man.*) sp.	Canopy						**1**					**0**	**0**	**0**	**1**	**N/A**
	Ground	1				**1**						**0**		**1**		
Sa. batesi	Canopy			1		**1**	**1**					**0**	**0**	**1**	**1**	**N/A**
	Ground											**0**		**0**		
Wy. sabethea	Canopy						**1**					**0**	**0**	**0**	**1**	**N/A**

	Ground	1				**1**						**0**		**1**		
Ae. aureolineatus	Canopy						**0**					**0**	**6**	**0**	**6**	**N/A**
	Ground							3			3	**6**		**6**		
Li. flavisetosus	Canopy						**0**					**0**	**5**	**0**	**5**	**N/A**
	Ground							2			3	**5**		**5**		
Sa. aurescens	Canopy						**0**					**0**	**5**	**0**	**5**	**N/A**
	Ground							3		2		**5**		**5**		
An. cruzii	Canopy						**0**	3		1		**4**	**4**	**4**	**4**	**N/A**
	Ground											**0**		**0**		
Wy. dyari	Canopy						**0**					**0**	**4**	**0**	**4**	**N/A**
	Ground							4				**4**		**4**		
Ru. Cerqueirai	Canopy						**0**					**0**	**3**	**0**	**3**	**N/A**
	Ground							2		1		**3**		**3**		
An. laneanus?	Canopy						**0**	1		1		**2**	**2**	**2**	**2**	**N/A**
	Ground											**0**		**0**		
Anopheles (*Ker*.) spp.	Canopy						**0**	1		1		**2**	**2**	**2**	**2**	**N/A**
	Ground											**0**		**0**		
Ae. fulvithorax	Canopy						**0**					**0**	**1**	**0**	**1**	**N/A**
	Ground							1				**1**		**1**		
Ru. humboldti	Canopy						**0**					**0**	**1**	**0**	**1**	**N/A**
	Ground										1	**1**		**1**		
Trichoprosopon spp.	Canopy						**0**					**0**	**1**	**0**	**1**	**N/A**
	Ground							1				**1**		**1**		
Wy. codiocampa	Canopy						**0**	1				**1**	**1**	**1**	**1**	**N/A**
	Ground											**0**		**0**		
Wy. aff. limai	Canopy						**0**		1			**1**	**1**	**1**	**1**	**N/A**
	Ground											**0**		**0**		
Limatus spp.	Canopy						**0**					**0**	**1**	**0**	**1**	**N/A**
	Ground							1				**1**		**1**		
**Total**		**648**	**429**	**394**	**466**		**1937**	**810**	**397**	**437**	**212**		**1856**		**3,79**	

[1]p-Value calculated using the Mann–Whitney U test with 5% significance ($p < 0.05$); N/A = not applicable.

Supplementary Table S4. Mosquito species tested for natural infection by YFV in Cariacica (CA), Pancas (PA), Venda Nova do Imigrante (VNa and VNb), and Santa Teresa (STa and STb) from February to June 2017.

Sampling/Local	Number of tested pools (Number of positive pools)													
	Adults Sampling 1								Adults Sampling 2		Immature Sampling		Total Tested Pools	Total Positive Pools
Species	CA	PA	STa	STb	VNa	VNb	MIR[1]	MLE[2]	PA	VNa	CA	VNa		
Hg. leucocelaenus	23 (4)	22 (0)	16 (1)	5 (2)	5 (3)	2 (1)	32.5	32.1	18 (0)	17 (0)			108	11
Hg. janthinomys/capricornii	2 (1)	2 (1)	2 (0)	5 (0)	1 (0)		54.1	35.8	5 (0)	7 (0)			24	2
Sa. chloropterus		3 (1)	2 (0)				62.5	43.6	7 (0)				12	1
Sa. identicus	4 (1)				2 (0)	2 (0)	38.5	26.4	1 (0)	1 (0)			10	1
Sa. soperi	33 (2)		1 (0)				12.3	12.1			1 (0)		35	2
Ae. aureolineatus	4 (0)		3 (1)	1 (0)	4 (0)		14.3	8.7		3 (0)			15	1
Sh. fluviatilis	5 (0)		1 (0)		9 (0)	4 (1)	5.9	5.4					19	1
Ae. fulvithorax					1 (0)		0	0					1	0
Ae. scapularis	1 (0)	16 (0)	21 (0)	4 (0)	2 (0)		0	0	6 (0)	1 (0)			51	0
Ae. Serratus Group	1 (0)	4 (0)	7 (0)	1 (0)	1 (0)	1 (0)	0	0	4 (0)				19	0
Ae. taeniorhynchus			1 (0)				0	0					1	0
Ae. terrens	1 (0)	2 (0)	3 (0)		4 (0)	1 (0)	0	0	1 (0)	8 (0)			20	0
Ae. aegypti			1 (0)				0	0					1	0
Ae. albopictus	2 (0)	10 (0)	3 (0)	12 (0)		1 (0)	0	0	7 (0)				35	0
Ps. albipes		9 (0)					0	0	2 (0)				11	0
Ps. albipes/albigenu/varipes		2 (0)					0	0					2	0
Ps. ferox	1 (0)	11 (0)	11 (0)	1 (0)		2 (0)	0	0	6 (0)	1 (0)			33	0
Ps. lanei			1 (0)			1 (0)	0	0					2	0
Sa. petrocchiae				4 (0)	1 (0)		0	0	4 (0)	2 (0)			11	0
Sa. aurescens	5 (0)				1 (0)		0	0		2 (0)			8	0
Sa. fabricii							0	0			4 (0)	1 (0)	5	0
Sa. undosus/fabricii	18 (0)		1 (0)		1 (0)		0	0			1 (0)		21	0
Sa. aff. *ignotus*	2 (0)						0	0					2	0
Sa. whitmani	2 (0)												2	0
Sa. albiprivus	2 (0)		3 (0)		5 (0)		0	0	4 (0)	6 (0)			20	0
Sa. batesi	1 (0)						0	0	1 (0)				2	0
Sa. forattinii							0	0	2 (0)				2	0
Sa. purpureus	2 (0)				1 (0)	1 (0)	0	0		9 (0)			13	0
Sa. intermedius	1 (0)		1 (0)				0	0					2	0

Sa. melanonymphe	2 (0)	1 (0)					0	0					3	0
Sa. xhyphydes	1 (0)	1 (0)	1 (0)				0	0					3	0
Wy. shannoni	2 (0)						0	0					2	0
Wy. lutzi	1 (0)	1 (0)	3 (0)	1 (0)	4 (0)	2 (0)	0	0					12	0
Wy. bonnei/deanei	1 (0)	3 (0)			2 (0)	5 (0)	0	0					11	0
Wy. aff. *davisi*	3 (0)	5 (0)	4 (0)		7 (0)	42 (0)	0	0					61	0
Wy. edwardsi	1 (0)	3 (0)	4 (0)		6 (0)	4 (0)	0	0					18	0
Wy. incaudata	9 (0)	4 (0)	4 (0)		3 (0)	4 (0)	0	0					24	0
Wy. pillicauda	2 (0)	3 (0)				2 (0)	0	0					7	0
Wy. incaudata/pilicauda					5 (0)	1 (0)	0	0					6	0
Wy. palmata/ galvaoi	2 (0)	6 (0)	3 (0)		5 (0)	25 (0)	0	0					41	0
Wy. confusa	1 (0)	1 (0)			9 (0)	4 (0)	0	0					15	0
Wy. bourrouli/forcipenis	5 (0)	14 (0)	3 (0)	2 (0)	2 (0)	1 (0)	0	0					27	0
Wy. mystes	7 (0)	5 (0)	3 (0)	1 (0)	5 (0)		0	0					21	0
Li. durhamii	26 (0)	32 (0)	4 (0)	1 (0)	3 (0)		0	0					66	0
Li. pseudomethysticus	34 (0)		15 (0)	1 (0)	4 (0)		0	0					54	0
On. personatum	4 (0)	1 (0)	3 (0)	6 (0)	5 (0)	4 (0)	0	0					23	0
Ru. frontosa	3 (0)	2 (0)	6 (0)		8 (0)	1 (0)	0	0					20	0
Tr. castroi/similis	23 (0)		3 (0)	1 (0)	12 (0)	2 (0)	0	0					41	0
Tr. pallidiventer	4 (0)	1 (0)	5 (0)		6 (0)	5 (0)	0	0					21	0
Total	241 (8)	164 (2)	139 (2)	46 (2)	124 (3)	117 (2)			68 (0)	57 (0)	6 (0)	1 (0)	963	19

[1]Minimum infection rate = number of YFV-positive pools / number of adults tested for this species × 1,000; [2]Maximum likelihood estimate per 1,000 mosquitoes = 1 − (1 – Y / X) ^ (1 / m), where Y is the number of positive pools, X is the total number of pools, and m is the size of each tested pool.

Supplementary Table S5. Number of YFV-positive mosquito pools correlated with the time elapsed between the first YFV record and the date of adult sampling 1 in Cariacica (CA), Pancas (PA), Venda Nova do Imigrante (VNa and VNb), and Santa Teresa (STa and STb), Espírito Santo, 2017.

Local	EW[1] of the 1st YFV record	EW of collection	Time elapsed between the 1st YFV record in human or/and NHPs and the beginning of mosquito collection date	Number of positive pools
VNa	3rd	6th	3 weeks	3
VNb	3rd	6th and 7th	3 weeks	2
PA	2nd	8th and 9th	6 weeks	2
STa	3rd	8th	5 weeks	2
STb	3rd	8th	5 weeks	2
CA	7th	9th	2 weeks	8

[1]EW (Epidemiological week, corresponding to the week of the year).
